# Meeting of the Fifth District Branch of the New York State Medical Society

**Published:** 1911-11

**Authors:** 


### Meeting of the Fifth District Branch of the New York State Medical Society

The Oneida County Society entertained the Fifth District Branch of the State Society on Thursday, October 5, at the New Century Club Auditorium, corner of Genesee and Hopper Streets, Utica.

The meeting was held in the club auditorium, luncheon being served in the auditorium parlors.

